# Viral regulation of organelle membrane contact sites

**DOI:** 10.1371/journal.pbio.3002529

**Published:** 2024-03-05

**Authors:** William A. Hofstadter, Elene Tsopurashvili, Ileana M. Cristea

**Affiliations:** Department of Molecular Biology, Princeton University, Princeton, New Jersey, United States of America

## Abstract

At the core of organelle functions lies their ability and need to form dynamic organelle–organelle networks that drive intracellular communication and coordination of cellular pathways. These networks are facilitated by membrane contact sites (MCSs) that promote both intra-organelle and inter-organelle communication. Given their multiple functions, MCSs and the proteins that form them are commonly co-opted by viruses during infection to promote viral replication. This Essay discusses mechanisms acquired by diverse human viruses to regulate MCS functions in either proviral processes or host defense. It also examines techniques used for examining MCSs in the context of viral infections.

## Abstract 2

Viruses are simple and rely on manipulating existing cellular structures to promote their replication. This Essay explores how viruses modulate different membrane contacts between organelles to promote viral replication, as well as the techniques used for studying membrane contact sites during viral infections.

## Introduction

A defining feature of eukaryotic cells is the compartmentalization of cellular functions. The subcellular space of eukaryotic cells is separated into organelles, which each have unique structures and compositions that are suited for their specific cellular roles. Traditionally, organelles have been studied as isolated entities that function independently of one another. However, advances in microscopy over the past 2 decades have revealed a highly complex picture of organelles that are associated in interconnected networks, giving rise to the field of membrane contact site (MCS) research [1–5]. MCSs are a close association (approximately 15 to 30 nm) of 2 or more organelles that allow rapid inter-organelle crosstalk without inducing membrane fusion. Organelle contacts are extensively found in yeast, plant, and animal cells, with every organelle, including membrane-less organelles, forming MCSs [6–8]. The hallmark roles of these associations include facilitating non-vesicular exchange of material (e.g., ions, lipids, and metabolites), fine-tuning organelle identity and dynamics, and regulating vesicle trafficking [3].

Organelle contact formation and function is controlled by several different classes of MCS proteins, which together give each MCS its distinct character [3]. Perhaps most simply, MCS proteins can serve structural roles, acting as tethers to connect organelles, or spacers to maintain a defined distance between organelle membranes [9]. MCS proteins can also serve more functional roles, facilitating the transfer of lipids, ions, or metabolites between organelles [10,11]. Furthermore, MCS proteins can regulate the identity and activity of a given MCS by recruiting other MCS proteins to a contact, altering the activity of proteins present at MCSs, or tuning the extent of contact [12]. A single protein can also serve several of the above listed roles. In fact, most functional MCS proteins also display some tethering ability [13].

Due to their essential roles in cellular homeostasis, organelle contacts are highly regulated. As such, MCS dysregulation is a hallmark of several neurodegenerative disorders, as well as genetic and metabolic diseases [14–18]. Similarly, viral infections can trigger reorganization of organelle contacts to modulate organelle structure and function, and to establish a successful replication cycle [19–27]. Viruses are obligate parasites, indicating that they must co-opt existing cellular pathways and modulate their homeostatic functions to instead support viral replication. In doing so, viruses must also be efficient, given that they encode relatively few proteins. Therefore, to maximize this limited coding capacity, viruses commonly dysregulate MCSs, given that these represent cellular communication points that control both organelle function and transport of molecules.

Diverse viruses are known to exploit MCSs to shift intracellular processes, including calcium signaling, lipid trafficking, and apoptotic and innate immune pathways, toward the benefit of the virus (Table 1). To accomplish this, viruses control MCS protein abundance, localization, posttranslational modifications, and protein–protein interactions [27–30]. In this Essay, we explore several of the core functions that MCSs can serve during viral replication, as well as providing specific examples of shared or striking virus-induced MCS modulations.

**Table 1:** Diverse viruses employ MCS proteins to remodel contacts.

MCS protein	Type of contact	Virus	Reference
DRP1	ER–mitochondria	DENV, influenza A	[31,32]
MAVS	ER–mitochondria	HCV, DENV	[29,31]
MFN2	ER–mitochondria	HIV-1	[33]
PTPIP51	ER–mitochondria	HCMV, HIV-1, influenza A	[27,32,34]
SYNJ2BP	ER–mitochondria	HIV	[35]
VAPB	ER–mitochondria, ER–peroxisome	HCMV, HCV	[27,36]
VDAC	ER–mitochondria	DENV, HCV, HIV-1, influenza A	[37–40]
VMP1	ER–mitochondria, endosome–ER, ER–LD	DENV	[41]
ANXA6	Endosome–ER	Influenza A	[42]
EMC4	Endosome–ER	SV40	[43]
EMC7	Endosome–ER	SV40	[43]
NPC1	Endosome–ER	HCV, influenza A, reovirus	[44–46]
ORP1L	Endosome–ER	Adenovirus	[47]
Rab7	Endosome–ER	SV40	[43]
TPC1	Endosome–ER	Ebola virus, MERS, Marburg virus	[24,48]
VAPA	Endosome–ER	HRV	[49]
OSPBs	ER–Golgi	Rhinoviruses, HCV, HRV	[49–51]
OSBPLs	ER–Golgi	Rhinoviruses	[50]
E-Syt1	ER–plasma membrane	HSV-1	[52]
ORAI1	ER–plasma membrane	Arenaviruses, Ebola virus, Marburg virus, HSV-1, HBV	[53–55]
STIM1	ER–plasma membrane	Lassa virus, Junín virus, Ebola virus, Marburg virus, HSV-1, HBV, rotavirus	[53–56]
BAP31	ER–mitochondria, ER–LD	Coronavirus	[57]
SAM	Mitochondria–LD	Coronavirus	[57]
ACBD5	ER–peroxisome	HCMV, HCV	[27,58]

DENV, dengue virus; ER, endoplasmic reticulum; HBV, hepatitis B virus; HCMV, human cytomegalovirus; HCV, hepatitis C virus; HIV-1, human immunodeficiency virus-1; HRV, human rhinovirus; HSV-1, herpes simplex virus type-1; LD, lipid droplet; MCS, membrane contact site; MERS, Middle East respiratory syndrome coronavirus; SV40, Simian vacuolating virus 40.

## MCSs as critical determinants of successful viral infections

Advances in the tools used to study MCSs have revealed how diverse viruses rely on MCS regulation for efficient replication. While MCSs can serve several critical roles, a common function for MCSs during viral replication is the movement of ions and lipids such as calcium and cholesterol [20,59]. Altering the flux of these metabolites can influence every step of the viral replication cycle, including entry, genome replication, assembly, and egress.

**Fig 1:**
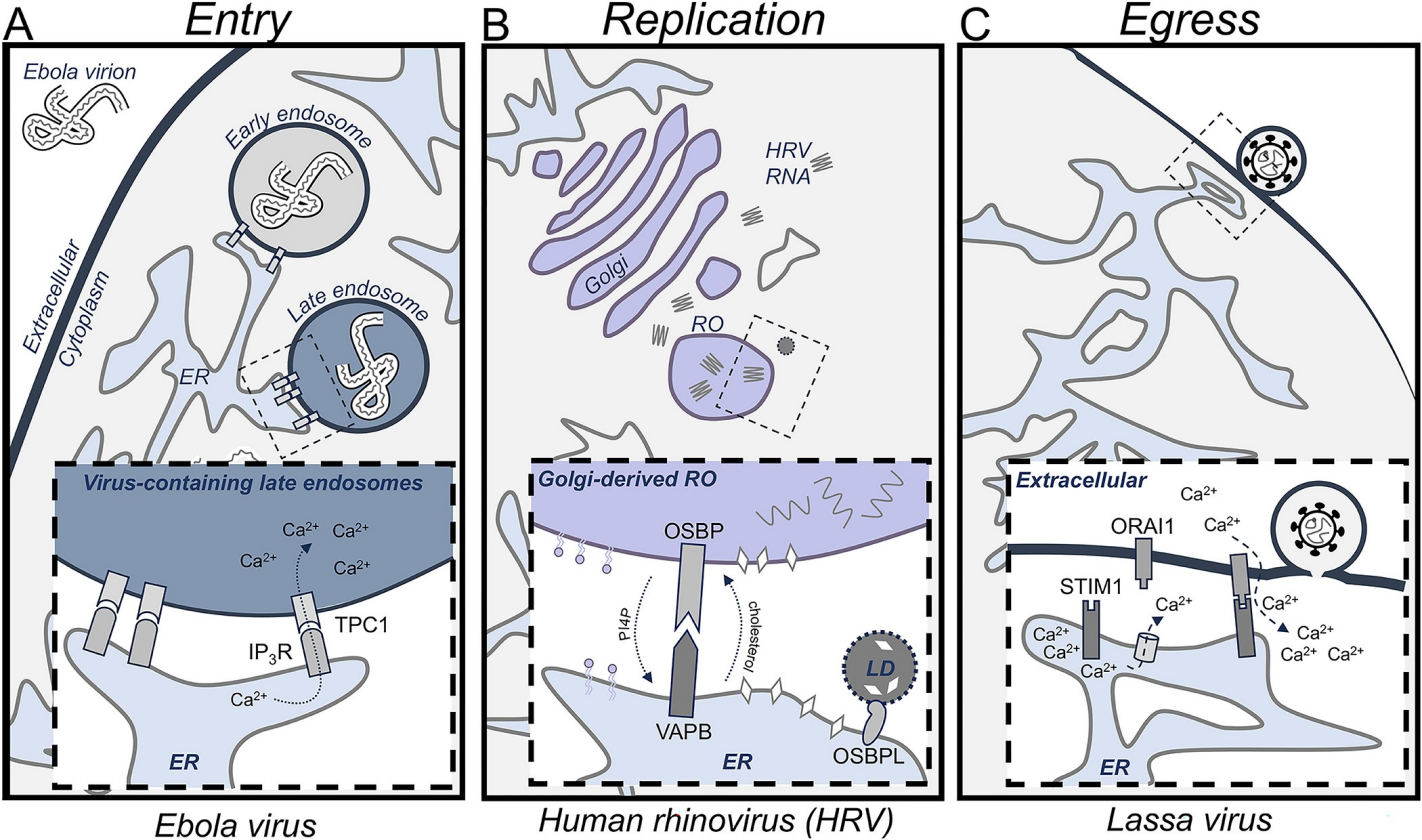
MCS alterations across the viral replication cycle. (**A**) Schematic of Ebola virus entry, which relies on IP_3_R and TPC1 ion channels (cutout). (**B**) HRV infection promotes cholesterol flux into Golgi-derived replication organelles (ROs) to promote genome replication. This is accomplished by the MCS proteins OSBP and VAPB, which mediate the exchange of PI4P from the Golgi for cholesterol from the ER and LDs (cutout). (**C**) Lassa virus opens extracellular calcium channels to promote virus egress. Lassa virus proteins induce the release of calcium (Ca2+) from the ER, which stimulates STIM1 to relocalize to ER–plasma membrane MCSs, together with ORAI1, to induce extracellular calcium import (cutout). ER, endoplasmic reticulum; HRV, human rhinovirus; LD, lipid droplet; OSBP, oxysterol-binding protein; RO, replication organelle.

A wide range of DNA and RNA viruses depend on endocytosis for entering cells and subsequent trafficking through the endolysosomal system for release into the cellular environment, processes that rely on host MCSs [60–62]. For example, 2 RNA viruses recently involved in epidemics, Ebola virus and Middle East respiratory syndrome coronavirus (MERS), require endosome-localized MCS proteins for release into cells [24,48]. These viruses rely on the acidic environment of the lysosome to promote uncoating and release of the virion (Fig 1A). In order to properly mature into a lysosome, endosomes must maintain contact with the endoplasmic reticulum (ER) [62]. To induce this maturation, Ebola virus and MERS target the MCS protein TPC1, which promotes calcium flux [63], to regulate the endosome–ER MCS (Table 1). Suppression of TPC1 impaired the ability of Ebola and MERS virions to travel through the endolysosomal system, which was sufficient to prevent Ebola virus infection in macrophages and to restrict MERS infectivity [24,48].

Following entry, viruses must replicate their genomes and assemble all virion components. Viruses have developed many different strategies for genome replication and assembly, depending on their genome type and virion structure. For example, most DNA viruses replicate in the host cell nucleus, where they can co-opt existing host replication machinery [64]. Alternatively, all positive-stranded RNA (+RNA) viruses replicate in an elaborate cytoplasmic web of membranous compartments called replication organelles that are enriched in the viral proteins and host factors needed for efficient genome replication [65]. Organelle contacts have emerged as critical host factors that are exploited by many viruses to generate and modulate replication organelles [66]. These pathogens have developed strategies for redirecting MCS proteins to the organelle contacts close to the replication sites to promote lipid flux for replication organelle biogenesis and maintenance [20,66,67].

Several +RNA virus families, including picornaviruses and rhinoviruses, remodel MCSs at the ER–Golgi interface to redirect lipid flow (Fig 1B). In an uninfected cell, oxysterol-binding proteins (OSBPs) control cholesterol transfer to the Golgi from the ER [68]. In doing so, OSBPs also metabolize the majority of phosphatidylinositol 4-phosphate (PI4P) lipids present at the Golgi membrane. Among others, rhinoviruses rely on this cholesterol–PI4P exchange to create cholesterol-enriched viral replication compartments [50] (Fig 1B and Table 1). Broadly, this is achieved by first increasing PI4P levels at the Golgi-derived replication organelle via one of the phosphatidylinositol 4 kinases. OSBP then mediates the exchange of PI4P for cholesterol to transport cholesterol to the replication organelle. To maintain a pool of cholesterol on the ER to be transported to the replication organelle, other MCS proteins, including OSBP-like (OSBPL) proteins, mediate lipid droplet and/or endosome MCSs with the ER at sites of viral replication. Highlighting the importance of these MCS-derived structures, knock-down of OSBP1 or expression of an OSBP1 mutant that lacks the ability to bind to PI4P, prevented the replication of several human rhinoviruses (HRVs) [50]. While most pervasive in +RNA viruses, the formation of replication organelle structures is also observed in some cytoplasmic-replicating DNA viruses, such as vaccinia virus [69].

Now assembled, the maturing virions must exit the cell via a process termed egress. For egress, viruses must again interface with the plasma membrane and endomembrane systems. Therefore, many of the MCS proteins involved in entry can also be rewired to promote viral egress. For example, several hemorrhagic fever viruses, including negative-stranded RNA (−RNA) arenaviruses and filoviruses, modulate the MCS proteins ORAI1 and STIM1 for egress [54], the same proteins that DNA virus herpes simplex virus type-1 (HSV-1) relies on for entry (Table 1) [53]. For the hemorrhagic fever viruses, viral matrix proteins stimulate ER calcium release, which then triggers STIM1 to relocalize to the plasma membrane and contact with ORAI1, thereby promoting the import of extracellular calcium into the cell (Fig 1C). This external calcium supply can then support many different aspects of viral replication, including promoting membrane fusion for entry or egress [70]. During HSV-1 infection, activation of this pathway leads to TRPC1 relocation to the plasma membrane, where it can bind to the viral glycoprotein gD, found on the surface of HSV-1 virions, and facilitate viral entry [53]. Alternatively, there are many egress specific tactics. Reovirus infection promotes the formation of viral inclusions, which are membranous structures where viral replication takes place [71]. These viral inclusions then use unknown MCS proteins to transfer mature virions into modified lysosomes, termed sorting organelles, which are subsequently targeted to the plasma membrane for non-lytic egress [71].

## MCSs and intrinsic immunity

**Fig 2:**
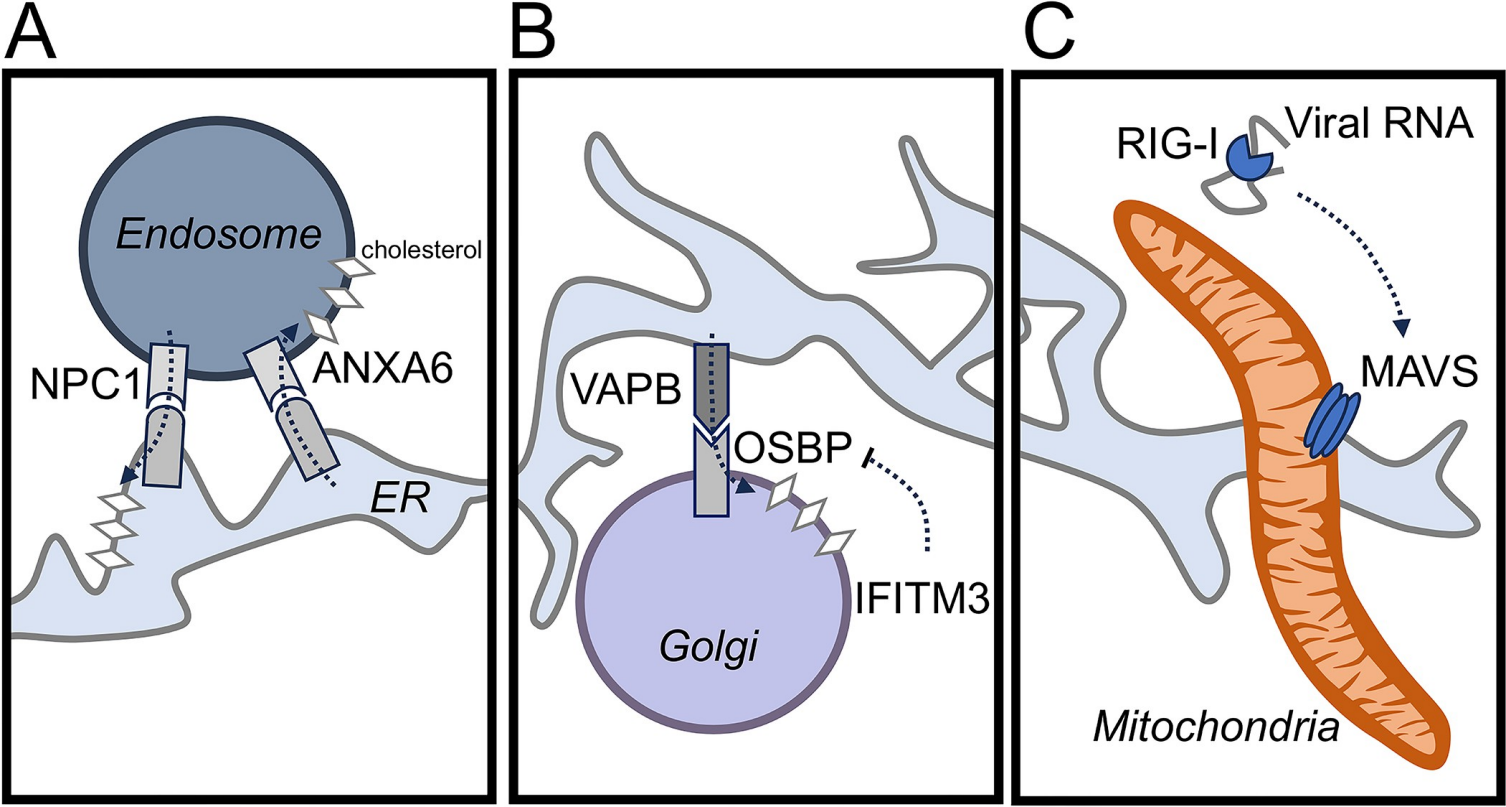
MCSs serve proviral and antiviral functions during infection. (**A**) Influenza A infection requires cholesterol enrichment at the plasma membrane for efficient replication. To reach the plasma membrane, cholesterol must first be trafficked out of endosomes and into the ER, a process which is promoted by the MCS protein NPC1 but countered by ANXA6. (**B**) Many viruses employ OSBP to flux cholesterol into the Golgi or Golgi-derived membranes. The antiviral protein IFITM3 functions to outcompete OSBP for binding to its partner VAPB, thus preventing cholesterol enrichment at the Golgi. (**C**) RIG-I recognizes cytosolic viral RNA and then localizes to ER–mitochondria MCS to activate MAVS and downstream antiviral signaling. ER, endoplasmic reticulum; MCS, membrane contact site; OSBP, oxysterol-binding protein.

While many viruses have developed unique methods for usurping MCSs throughout their replication cycles, it is important to remember that the host cell is not defenseless. Once viral replication is detected within a host cell, several intrinsic immune signaling pathways have evolved mechanisms of shutting down commonly subverted MCS proteins. One way through which the host cell does this is by activating MCS proteins that are antagonistic to the needs of the virus. For example, the enveloped RNA virus influenza A requires cholesterol enrichment at the plasma membrane for virion assembly, a process that is promoted by the MCS protein NPC1 (Table 1) [45]. NPC1 functions to mobilize cholesterol out of endosomes and into the ER, where downstream MCS proteins can then transfer it to the plasma membrane (Fig 2A). To counteract this flow, the host cell employs the MCS protein ANXA6, which instead promotes the accumulation of cholesterol in endosomes (Table 1) [42]. This is a critical stage in influenza A infection, given that suppression of NPC1 activity or overexpression of ANXA6 prevents viral assembly [42,45]. Similarly, cholesterol loading can prevent the trafficking of vesicular stomatitis virus protein G to the plasma membrane [72]. Therefore, in addition to co-opting MCS functions, viruses must also subvert the antiviral activities of some MCS proteins.

Alternatively, host cells may more directly block the function of a proviral MCS proteins. As mentioned, cholesterol redistribution is a common requirement for viral infection, and therefore, represents a broad-spectrum target for antiviral signaling pathways. This is exemplified by IFITM3, which is activated in response to diverse viral infections and exerts one of its antiviral functions by disrupting proviral MCS protein interactions [28]. When stimulated, IFITM3 outcompetes OSBP for binding to VAPA, which subsequently dysregulates cholesterol cycling (Fig 2B). The resultant accumulation of cholesterol traps the invading virions in multivesicular bodies, preventing viral replication before it starts [73]. Further indicating that this step is a common lynchpin in viral replication, antiviral signaling pathways also promote oxidation of cholesterol, forming such oxysterols as 25-hydroxycholesterol and 27-hydroxycholesterol. These chemicals exhibit broad-spectrum antiviral activities against diverse DNA, RNA, enveloped and non-enveloped viruses by altering membrane lipid compositions and thus suppressing membrane fusion [74,75].

Just as host cells have developed ways of suppressing proviral MCS functions, viruses have also developed to use MCSs to dampen antiviral signaling pathways. One common target for suppressing antiviral pathways is the mitochondria, which in addition to being the main energy producer for the cell, also acts as a central hub for many immune signaling axes. For example, RNA viruses can be detected by the host cell via the cytoplasmic sensor RIG-I, which in turn activates MAVS at ER–mitochondria MCS to ultimately induce an immune response [29,76] (Fig 2C). During hepatitis C virus (HCV) infection, the viral protein NS4A localizes specifically to ER–mitochondria MCSs to cleave MAVS and stunt this signaling pathway (Table 1) [27–29]. Alternatively, during dengue virus infection, the viral protein NS4B prevents the activation of host mitochondrial fission-factor Drp1 (DNM1L in humans), resulting in increased MCSs between mitochondria and virus-induced replication organelles (Table 1) [31]. This modulation leads to a concurrent decrease in ER–mitochondria MCSs, and thus dampens MAVS activation [31]. Conversely, viral proteins can directly suppress mitochondrial membrane potential, which is required for MAVS activation [77]. Pandemic strains of influenza A employ the viral protein PB1-F2 to form MCSs between the inner and outer mitochondria membranes, resulting in the formation of the permeability transition pore complex and subsequent membrane depolarization [40,77]. This is a particularly useful strategy for viruses that replicate quickly and do not require energy production from mitochondria.

## Virus-induced MCS dysregulation remodels the cellular landscape

MCS proteins help to give individual organelles their identity, while also facilitating inter-organelle communication. Hence, MCS protein dysregulation during viral infection promotes many of the observed changes to the organelle landscape, as well as many of the hallmark features of particular infections.

**Fig 3:**
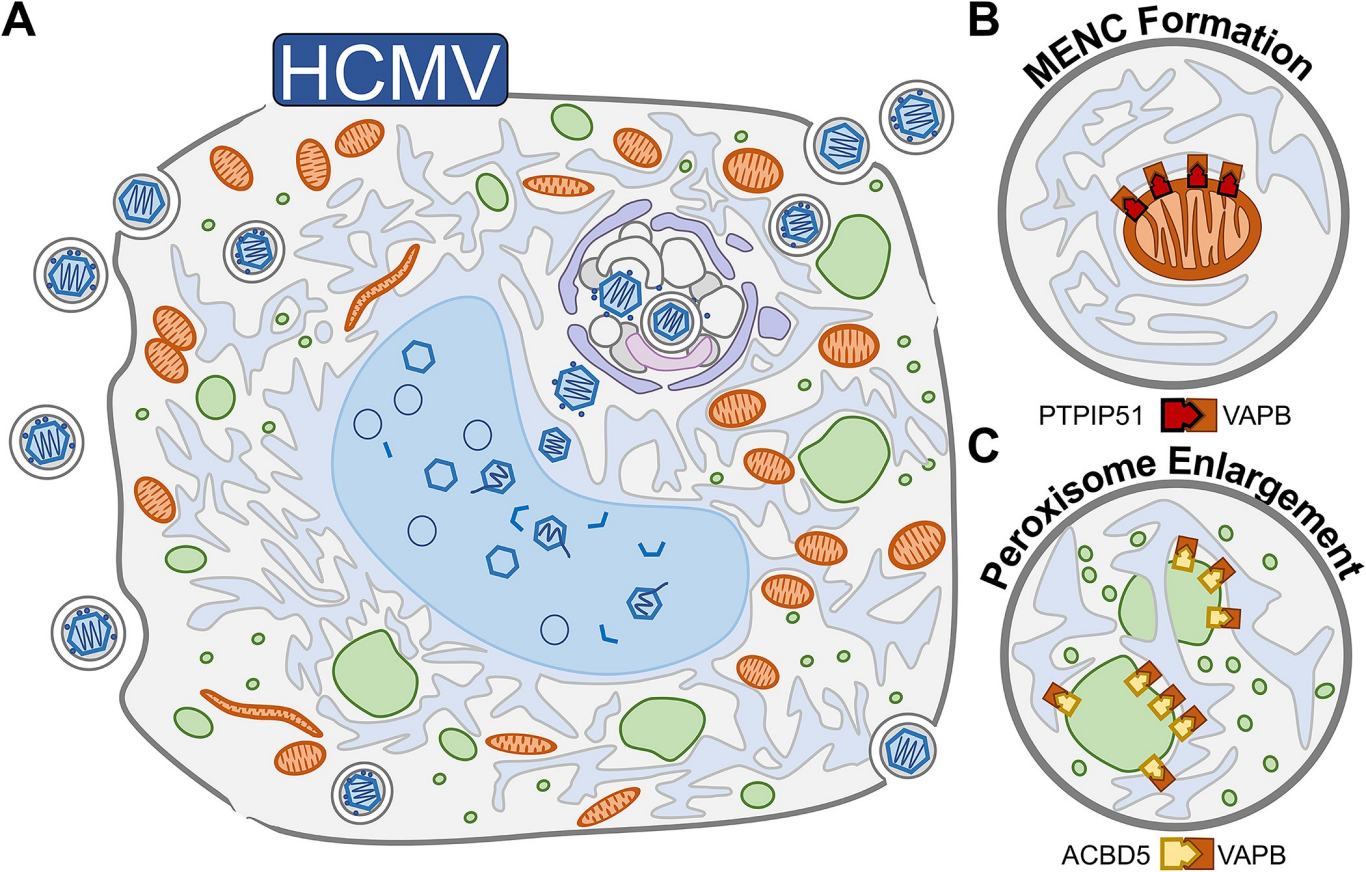
HCMV modulates organelle structure and function by co-opting MCSs. (**A**) HCMV remodels nearly every organelle during infection, including mitochondria (orange), the nucleus (blue), peroxisomes (green), the ER (light blue), and Golgi/endosomes (purple). (**B**) MENCs mediated by PTPIP51 and VAPB become the dominant ER–mitochondria MCSs by late stages of HCMV infection. (**C**) HCMV also promotes the formation of a subset of enlarged and irregularly shaped peroxisomes using the MCS proteins ACBD5 and VAPB. ER, endoplasmic reticulum; HCMV, human cytomegalovirus; MCS, membrane contact site; MENC, mitochondria–ER encapsulation.

One striking example of MCS-induced organelle remodeling is during infection with human cytomegalovirus (HCMV; Fig 3A). This large DNA virus replicates over a lengthy 120-h period, during which viral proteins must accomplish the dual roles of generating enough energy for viral replication and keeping the host cell alive until virion egress. To achieve this, HCMV infection up-regulates nearly all MCS protein abundances throughout the viral replication cycle [27], concurrent with the remodeling of every subcellular organelle [26,78,79]. In particular, HCMV infection remodels ER–mitochondria MCSs to form structures termed mitochondria–ER encapsulations (MENCs) [27]. MENCs consist of a temporally stable, asymmetric cupping of mitochondria by ER tubules (Fig 3B). The MCS proteins VAPB and PTPIP51 localize to MENCs during infection and are necessary for efficient viral replication (Table 1) [27]. While the function of this contact remains unclear, MENCs are well situated to promote calcium [11,80] and lipid [81] exchange or to prevent mitochondria degradation [82] in order to promote mitochondrial activity.

VAPB establishes inter-organelle tethers with many different MCS proteins, forming contacts between the ER and diverse organelles. For example, HCMV also leverages VAPB to promote ER–peroxisome contacts through an interaction with peroxisomal protein ACBD5 [27] (Fig 3C and Table 1). ACBD5 and VAPB help to coordinate the synthesis of plasmalogen lipids [83], which is initiated in peroxisomes and finalized in the ER, as well as to promote peroxisome enlargement [27]. Consequently, throughout HCMV infection, there is an increase in plasmalogen synthesis in conjunction with the formation of a subset of enlarged and irregularly shaped peroxisomes [84]. Similarly, ACBD5 has been implicated in forming enlarged peroxisomes at HCV replication organelles (Table 1) [58]. Plasmalogens are necessary for secondary envelopment during HCMV virion assembly [84] and are enriched in the envelope of HCMV virions [85]. Somewhat surprisingly, both ACBD5 overexpression and knockdown decrease HCMV replication efficiency [27]. This finding highlights the need for viruses to temporally tune MCS protein abundance across their replication cycle.

While HCMV is a good example of a virus that rewires MCSs to increase the productivity of the host cell, many other viruses that replicate over shorter time frames instead employ MCSs to induce host cell death. Viruses including poliovirus, human immunodeficiency virus-1 (HIV-1), and influenza A promote host cell death by regulating the abundance of calcium channels (Table 1) [39,40,86]. All of these viruses stimulate the sudden influx of calcium from the ER into mitochondria via the MCS proteins VDACs [59]. This rapid influx of calcium results in mitochondrial collapse and subsequent activation of intrinsic apoptosis pathways. Given that this mechanism is conserved across several viral families, and that virus-induced cell death contributes to pathogenesis, blocking the interactions between viral proteins and VDACs could lead to a potent broad-spectrum antiviral response [39]. Intriguingly, many viral proteins first localize to ER–mitochondria MCSs prior to promoting mitochondria dysfunction [33,87]. This further illustrates how MCSs act as control centers for modulating many different organelle functions.

## Method improvements for MCS characterization during virus infection

Technological advancements have allowed us to identify, quantify, and characterize many new membrane contacts with greater precision than ever before. In 1956, electron microscopy (EM) was the first technology that allowed for the visualization of MCSs [88]. Since then, advancements in EM and electron tomography (ET) have allowed researchers to directly visualize MCSs between organelles and to quantify several parameters of an organelle contact, such as distance between membranes, number of contacts, and length of contact [3,89,90]. EM has also been used to identify many hallmark features of viral infections involving MCSs, including the formation of replication organelles, such as the double membrane vesicles formed during coronavirus infections [91].

The cellular environment is intrinsically crowded and complex, which can make it difficult to identify specific structures within an EM image. To combat this, correlative light and electron microscopy (CLEM) can be used. CLEM is achieved by imaging fluorescently labeled samples with both light microscopy and EM, then integrating these 2 images to harness the specificity of light microscopy with the resolution of EM [63,92]. This technique is particularly useful during viral infections where the subcellular landscape is dramatically remodeled, which can erase common landmarks seen in uninfected cells. Furthermore, CLEM can leverage the ability of light microscopy to visualize the functions of MCSs. For example, CLEM was used to show that, during HCV infection, the viral protein NS5A colocalizes with sites of MCS-dependent cholesterol flux, which are used to form the replication organelle [44]. Alternatively, advancements in machine learning and artificial intelligence have allowed for the automated identification, segmentation, and quantification of MCSs in EM images [93,94].

While light microscopy cannot compete with the resolution of EM, this technique is much easier and more economical to perform, while also supporting the imaging of live cells. Moreover, new technologies have pushed the limits of what is possible with light microscopy, making this technique an attractive alternative. For example, advances in spectral imaging allow researchers to concurrently monitor over a dozen fluorescent probes. Spectral imaging can subsequently be leveraged for screening many different MCSs in a single sample [95]. A myriad of advances in super-resolution microscopy have also allowed for visualization of MCSs with increased temporal and spatial resolution [96,97]. In a recent study, 3D super-resolution microscopy was integrated with deep learning to explore how Zika virus proteins remodel organelles. The viral protein NS4B was shown to disrupt MCSs between the ER and mitochondria, contributing to subversion of host intrinsic immune signaling [98].

The intrinsic qualitative nature of microscopy has been complemented by advancements in quantifying MCS frequency in both fixed and live samples. For example, in a proximity ligation assay (PLA) [99], a specific fluorescent signal is generated if antibodies added to fixed cells are in close proximity (<40 nm). This allows for quantification of the localization and frequency of endogenous protein–protein interactions present at MCSs. Therefore, while light microscopy may not have the resolution to determine if 2 organelles are within the defined MCS range of 15 to 30 nm, PLA can confirm this, as well as identify the specific proteins contributing to that MCS. This powerful tool was applied during HCMV infection to confirm increased ER–peroxisome and ER–mitochondria MCSs [27]. Alternatively, split fluorescent proteins can be used to quantify MCSs in live cells [100]. In this assay, 2 plasmids are designed to each have a localization signal for a particular organelle of interest, as well as complementary halves of a split fluorescent protein such as GFP. Therefore, when these constructs are in close proximity, a quantifiable signal is generated by the reformation of the fluorescent protein.

In addition to quantifying the abundance and localization of MCSs, there is significant interest in identifying the different proteins present at a given MCS to better ascertain their function. One efficient tool for answering this question is a split BioID method, termed ContactID [101]. BioID is an engineered biotin ligase that biotinylates proteins in its near vicinity [102]. Biotinylated proteins can then be extracted and characterized by proteomics. By splitting the BioID, the biotin ligase is only activated at sites where 2 organelles of interest are in close proximity, in a similar manner to the split fluorescent system. In the context of infection, ContactID also has the capacity to identify how viral proteins may be interfacing with a given MCS.

The drawback to many of the above assays is that they require a priori knowledge of which MCS proteins are of interest in a sample. Additionally, many MCS proteins are present at a low abundance and lack good/affordable antibodies for profiling them. Targeted mass spectrometry can be used to profile the abundances of all MCS proteins throughout an infection to identify which contacts and proteins are worth investigating. Given that MCS protein abundance can be used as an indicator of the extent of contact [103], this assay is a powerful tool for identifying alterations to MCSs at a systems level. Recently, an assay using the targeted mass spectrometry method of parallel reaction monitoring (PRM) was developed to simultaneously quantify the abundance of nearly all known MCS proteins in human cells [27]. This method was applied to monitor MCS proteins across infections with HCMV, HSV-1, influenza A, and the coronavirus OC43. When combined with microscopy and functional assays, this analysis [27] showed that these diverse viruses differentially regulate MCSs on a granular and cellular scale.

**Fig 4:**
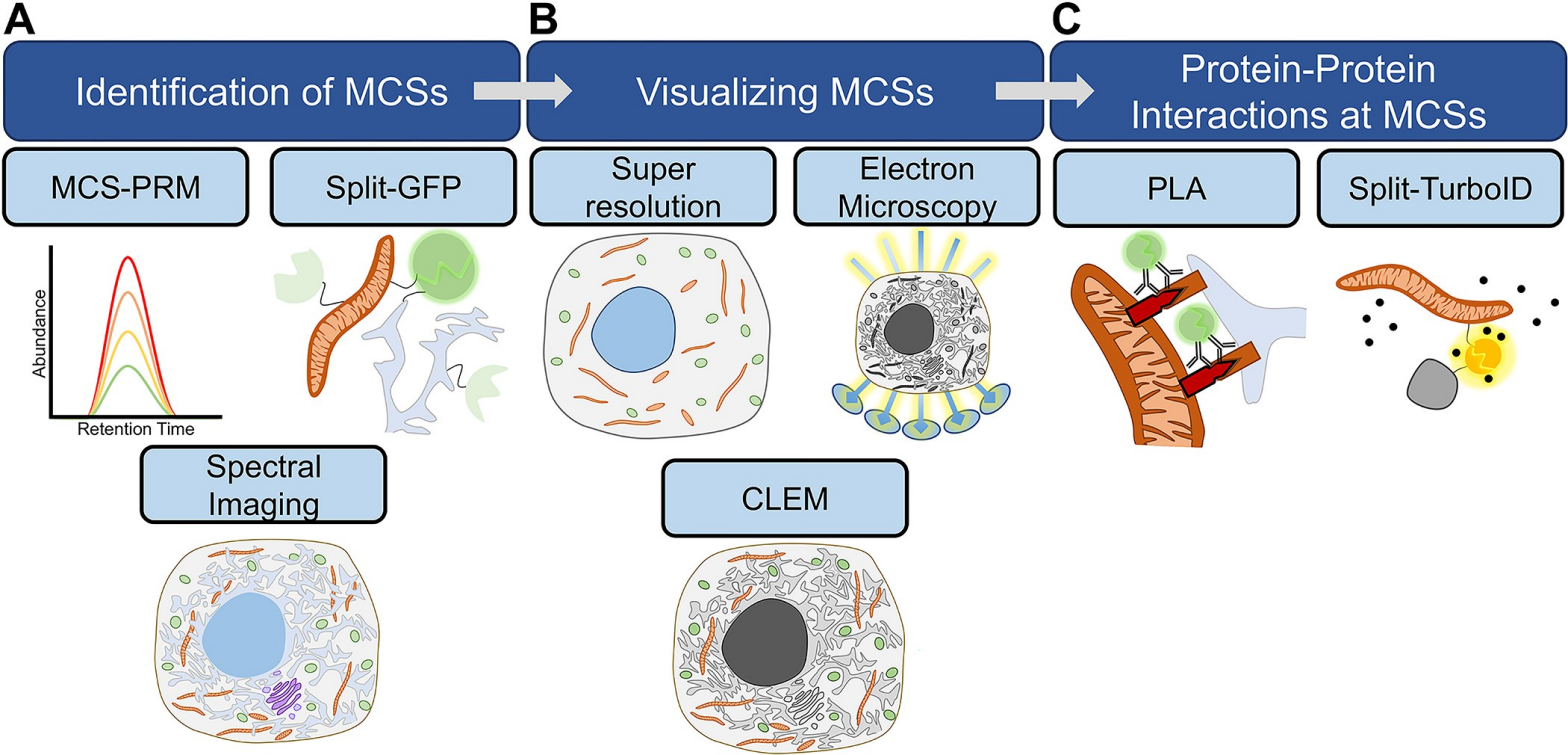
Proposed workflow for MCS identification and characterization. (**A**) MCSs can first be identified using MCS–PRM, which uses targeted mass spectrometry to quantify MCS protein abundances across a virus replication. Alternatively, numerous split-fluorescent probes or spectral imaging can be used to simultaneously monitor MCS formation. (**B**) MCSs should be visualized at high resolution, using super-resolution confocal microscopy techniques, EM/tomography or, ideally, combining the 2 to perform CLEM. (**C**) The protein–protein interactions that form an MCS of interest can be visualized using a PLA, while a split-TurboID system (ContactID) can identify the proteins that localize to MCSs. CLEM, correlative light and electron microscopy; EM, electron microscopy; MCS, membrane contact site; PLA, proximity ligation assay; PRM, parallel reaction monitoring.

When taken together, these tools allow researchers to identify, quantify, and assign functionalities to new MCSs, as well as to characterize MCS perturbations in diverse disease states. While several examples of virus-mediated MCS alterations have been provided in this Essay, there is still much to discover in this growing field given the abundance of both viral pathogens and MCSs. We therefore propose a workflow for the identification and characterization of MCSs of interest during any viral infection (Fig 4). First, a screening method can be used to identify MCSs of interest (Fig 4A). MCS–PRM is an efficient method for simultaneously monitoring most MCSs and the proteins that comprise them across a viral replication cycle. If access to a mass spectrometer is limited, split fluorescent probes targeted to different organelles coupled with spectral imaging can instead be used to simultaneously monitor MCSs. Second, higher resolution imaging should be employed to visualize the MCSs identified in the first step (Fig 4B). This is particularly important as a follow-up to MCS–PRM. Next, PLA can be used to identify the protein–protein interactions mediating MCSs of interest (Fig 4C). Finally, a split-TurboID system coupled with mass spectrometry can be leveraged to identify the proteins present at an MCS of interest, which can inform on the functionality of the MCS as well as potentially identify the viral proteins responsible for modulating it. This workflow can aid in expanding the understanding of infection-induced remodeling of organelle–organelle contacts and the possible coordinated functions of MCSs in virus replication and host defense.

## Conclusion and future directions

Despite the diverse array of techniques available for studying MCSs and the importance of these tethers in the context of viral infection, many facets of this field remain unexplored. MCSs have traditionally been selected for characterization during viral infections based on a priori knowledge, such as regarding an organelle remodeling event (e.g., replication organelle formation) or altered flux of a small molecule (e.g., calcium signaling). The implementation of unbiased studies, such as using a mass spectrometry approach (e.g., MCS–PRM), can widen the scope of MCSs monitored throughout viral infections. It is worth considering that many of the powerful techniques previously used to study MCSs, such as ContactID [101], are yet to be applied to viral infection studies. This method has the potential to reveal mechanistic insight into viral replication strategies, particularly given that there is still limited information about how viral proteins are specifically inducing the observed changes to MCSs. One underexplored way through which viral proteins may regulate MCSs is by directly engaging host MCS proteins to form novel tethers. This could be further explored by analyzing viral proteomes for common interaction motifs found in MCS proteins, such as the FFAT domain that facilitates an interaction with VAPA/VAPB [104].

Identification of MCSs and MCS proteins targeted during an infection is critical for several reasons. First, given that MCS are lynchpins for many viral infections and that different viruses target similar MCSs for their replication, screening for affected contacts has the potential to point to novel drug targets and possibly broad-spectrum antiviral therapeutic interventions. Second, since their original discovery, the study of viruses has led to many fundamental findings about basic biology [105]. This continues to be true today and includes the study of MCSs during viral infection. Much remains to be discovered about the formation, function, and regulation of MCSs in uninfected cells. Given that viruses modulate the existing host infrastructure, viral infections have the potential to amplify normal subtle features of MCSs, revealing their importance inside and outside the context of infection. Hence, the continued study of MCS alterations during viral infection is expected to offer an important perspective for understanding both virus and host biology.

## Abbreviations

CLEM: correlative light and electron microscopy
EM: electron microscopy
ER: endoplasmic reticulum
ET: electron tomography
HCMV: human cytomegalovirus
HCV: hepatitis C virus
HIV-1: human immunodeficiency virus-1
HRV: human rhinovirus
HSV-1: herpes simplex virus type-1
MCS: membrane contact site
MENC: mitochondria–ER encapsulation
MERS: Middle East respiratory syndrome coronavirus
OSBP: oxysterol-binding protein
PLA: proximity ligation assay
PI4P: phosphatidylinositol 4-phosphate
PRM: parallel reaction monitoring
